# Retest reliability of repetitive transcranial magnetic stimulation over the healthy human motor cortex: a systematic review and meta-analysis

**DOI:** 10.3389/fnhum.2023.1237713

**Published:** 2023-09-13

**Authors:** Carolina Kanig, Mirja Osnabruegge, Florian Schwitzgebel, Karsten Litschel, Wolfgang Seiberl, Wolfgang Mack, Stefan Schoisswohl, Martin Schecklmann

**Affiliations:** ^1^Institute of Psychology, University of the Bundeswehr Munich, Neubiberg, Germany; ^2^Department of Psychiatry and Psychotherapy, University of Regensburg, Regensburg, Germany; ^3^Department of Electrical Engineering, University of the Bundeswehr Munich, Neubiberg, Germany; ^4^Institute of Sport Science, University of the Bundeswehr Munich, Neubiberg, Germany

**Keywords:** neuromodulation, cortical excitability, rTMS, variability, motor evoked potentials, reliability, protocols

## Abstract

**Introduction:**

Repetitive transcranial magnetic stimulation (rTMS) is used to induce long-lasting changes (aftereffects) in cortical excitability, which are often measured via single-pulse TMS (spTMS) over the motor cortex eliciting motor-evoked potentials (MEPs). rTMS includes various protocols, such as theta-burst stimulation (TBS), paired associative stimulation (PAS), and continuous rTMS with a fixed frequency. Nevertheless, subsequent aftereffects of rTMS are variable and seem to fail repeatability. We aimed to summarize standard rTMS procedures regarding their test–retest reliability. Hereby, we considered influencing factors such as the methodological quality of experiments and publication bias.

**Methods:**

We conducted a literature search via PubMed in March 2023. The inclusion criteria were the application of rTMS, TBS, or PAS at least twice over the motor cortex of healthy subjects with measurements of MEPs via spTMS as a dependent variable. The exclusion criteria were measurements derived from the non-stimulated hemisphere, of non-hand muscles, and by electroencephalography only. We extracted test–retest reliability measures and aftereffects from the eligible studies. With the Rosenthal fail-safe N, funnel plot, and asymmetry test, we examined the publication bias and accounted for influential factors such as the methodological quality of experiments measured with a standardized checklist.

**Results:**

A total of 15 studies that investigated test–retest reliability of rTMS protocols in a total of 291 subjects were identified. Reliability measures, i.e., Pearson's *r* and intraclass correlation coefficient (ICC) applicable from nine studies, were mainly in the small to moderate range with two experiments indicating good reliability of 20 Hz rTMS (*r* = 0.543) and iTBS (*r* = 0.55). The aftereffects of rTMS procedures seem to follow the heuristics of respective inhibition or facilitation, depending on the protocols' frequency, and application pattern. There was no indication of publication bias and the influence of methodological quality or other factors on the reliability of rTMS.

**Conclusion:**

The reliability of rTMS appears to be in the small to moderate range overall. Due to a limited number of studies reporting test–retest reliability values and heterogeneity of dependent measures, we could not provide generalizable results. We could not identify any protocol as superior to the others.

## 1. Introduction

Transcranial magnetic stimulation (TMS) is a non-invasive brain stimulation (NIBS) technique with which brain activity can be induced and modulated. By applying TMS via single pulses (spTMS), momentary states of cortical excitability can be assessed; for example, stimulations over the motor cortex can induce a motor-evoked potential (MEP) in the respective contralateral hand muscle (Rossini and Rossi, 1998). TMS applied in a repetitive manner (rTMS) is deployed with either a fixed frequency with common frequencies of 1, 10, or 20 Hz or with complex patterns to induce longer-lasting neuroplastic changes (aftereffects) in the brain (Hallett, 2000; Siebner and Rothwell, 2003). MEPs are also used to capture these aftereffects qualitatively. Literature suggests that changes in cortical excitability after rTMS are depending on stimulation frequency (Fitzgerald et al., 2006). Hereby, frequencies at approximately 1 Hz lead to the inhibition of neuronal activity, i.e., lower MEPs after rTMS than before, and stimulation with frequencies over 5 Hz evoke facilitatory aftereffects, i.e., higher MEPs after rTMS than at the baseline. This assumption is referred to as the low-frequency inhibitory–high-frequency excitatory (lofi-hife) heuristic (Prei et al., 2023). Common patterned rTMS procedures are paired associative stimulation (PAS), whereby electrical stimulation of the respective peripheral muscle (conditioning stimulation; CS) is applied in close relation to the contralateral TMS pulse, and theta-burst stimulation (TBS), whereby triplets at 50 Hz are repeatedly delivered with a 5-Hz inter-burst-interval. The latter can be administered as intermittent TBS (iTBS) with a pause of 8 s between 2 s of stimulation or as continuous TBS (cTBS) without breaks. Regarding aftereffects, cTBS is considered to elicit inhibitory effects as well as PAS with an inter-stimulus interval (ISI) of 10 ms between CS and TMS pulse (PAS_10_), whereas iTBS and PAS with an ISI of 25 ms (PAS_25_) tend to be excitatory (Huang et al., 2005; Wischnewski and Schutter, 2016). All of these procedures have been frequently used in both basic and clinical research settings as well as in the treatment of various neurological and psychiatric disorders (Berlim et al., 2013, 2017; Patel et al., 2020; Shulga et al., 2021). However, recent literature shows high inter- and intra-subject variability in rTMS aftereffects, questioning the heuristics of a clear association of inhibition or facilitation with a specific protocol (Fitzgerald et al., 2006).

The variability of TMS and rTMS outcome parameters has been a major topic in the NIBS community (Guerra et al., 2020; Goldsworthy et al., 2021). Furthermore, with high variability of rTMS aftereffects, their reliability can decrease. As one of the three main quality criteria of scientific experiments, test–retest reliability is important because it indicates whether a measurement or intervention is precise and can be repeated over time while generating the same output. Reliability is most commonly measured with Pearson's *r* (*r*) and intraclass correlation coefficient (ICC), but both measures are not always reported. Moreover, there is no consensus on whether rTMS reliability is assessed for the measurements of MEPs after rTMS only (post) or rTMS aftereffects (MEPs from before rTMS application subtracted from MEPs after rTMS). To date, the focus of rTMS research has been primarily on the identification and enhancement of aftereffects, but whether these effects are reproducible over time has been rather neglected. With this review, we aimed to give an overview and classification of test–retest reliability of rTMS procedures. We investigated whether there is a most effective protocol to reliably induce neuroplastic changes in the brain. We conducted a meta-analysis with moderator variables to exclude that study-inherent parameters influence the reliability outcome. Hereby, we refer to parameters that can influence aftereffects of NIBS and their variability and depend on the equipment and schedule of laboratories and that cannot be adjusted throughout the experiment. These parameters we wanted to account for are the use of neuronavigation (Herwig et al., 2001; Julkunen et al., 2009), sex of participants (Pitcher et al., 2003), repetition interval (Hermsen et al., 2016), year of publication, excitability of protocol, and methodological quality of the study. To assess methodological quality, we chose the checklist by Chipchase et al. (2012). The authors created a checklist via the Delphi procedure that assesses whether publications descriptively “report” and experimentally “control” for participant factors, methodological factors, and analytical factors, which are observed as likely to influence MEP responses elicited by TMS. With the percentage of applicable items from the checklist, one has an approximate measure of the overall methodological quality of a study ranging from 0 to 100%. To validate our assessment, we checked for interrater agreement. Moreover, we have provided an overview of rTMS aftereffects for each of the protocols within the studies assessing reliability. In order to identify the most effective protocol to induce neuroplastic changes, we compared these protocol-specific aftereffects. Publication bias was examined to assess whether the reliability values of current studies are representative.

## 2. Methods

### 2.1. Inclusion criteria

Author CK performed a literature search in PubMed (latest in March 2023) using the keywords (“rTMS” OR “TBS” OR “theta burst” OR “PAS” OR paired asso^*^ stim^*^ OR repet^*^ transc^*^ magn^*^ stim^*^) AND (MEP OR motor evoked potentials OR cort^*^ exci^*^ OR plast^*^) AND (reli^*^ OR reproduc^*^ OR repeat^*^). No filters or automation tools were applied. Based on the PRISMA flow diagram schema (Page et al., 2021), we extracted articles with experiments that applied (1) any kind of rTMS, TBS, or PAS (2) the technique at least twice (3) stimulation area over the motor cortex (4) investigating healthy subjects (5) with measurements of MEPs via spTMS as a dependent variable. As target muscles, we focused on hand muscles to ensure comparability. In the first step, author CK screened records in the PubMed database by title and abstract. Second, authors CK and MO inspected independently the full text for the abstracts screened to confirm eligibility and inclusion in the review. The exclusion criteria were (1) measures derived from the non-stimulated hemisphere, i.e., different stimulation locations of rTMS and spTMS, (2) examination of non-hand muscles only, and (3) reports of only electroencephalography measurements. Other search strings did not manage to find all the articles we had included.

### 2.2. Test–retest reliability

For interpreting reliability and conducting the meta-analysis, we only used the studies in which a reliability measure such as ICC or *r* was conducted. Moreover, whenever two dependent measures of the identical session were made or both ICC and *r* values were calculated, we only used one value each for depiction and within the meta-analysis in order to prevent overrepresentation. Preferably, we extracted the *r* value over the whole measurement because it has a fixed range of values, whereas ICC calculations need to be correctly selected and reported to exclude biases. Nevertheless, in the summarizing Table 1, all reliability values are listed.

**Table 1:** Overview of included studies and values of interest.

**References**	**Protocol**	**N**	**Test**	**Reliability value**	**Repetition in days**	**Observed excitability (rTMS aftereffect)**	**Sex ratio (f/m)**	**NN**
Fratello et al. (2006)	PAS_25_	18	ICC	APB (post): **0.7**	7	1) **+36.4%**, 2) **+31.7%**	1	No
				ADM (post): 0.62				
				APB (post-pre): 0.05				
				ADM (post-pre): −0.003				
Sale et al. (2007)	PAS_25_	10	ICC	Long: **0.68**	7	Long: **+11%**	1.55	No
	Long, short	10		Short: **0.29**		Short: **+51%**		
	Morning,			Morning: 0.39				
	Afternoon			Afternoon: 0.71				
				Long (morning): 0.296				
				Long (afternoon): 0.513				
				Short (morning): 0.011				
				Short (afternoon): 0.812				
Boucher et al. (2021)	iTBS,	24	ICC	iTBS (*r*): **−0.284**	30	iTBS: **+14.18%**	0.5	Yes
	cTBS,		+ *r*	cTBS (*r*): **0.298**		cTBS: **+5.49%**		
	Sham			Sham (*r*): **0.149**		Sham: **+18.1%**		
				iTBS (ICC): [−0.22;−0.05]				
				cTBS (ICC): [−0.23;0.51]				
				sham (ICC): [−0.14;0.42]				
Jannati et al. (2019)	cTBS	28	ICC	AUC: **0.4**	9.5	1) –**50%**, 2) –**53%**	0.12	Yes
				|AUC|: 0.46				
Hinder et al. (2014)	iTBS	30	ICC	ICC: 0.534	7	**+30.2%**	1.73	Yes
			+ *r*	*r*: **0.55**				
Schilberg et al. (2017)	iTBS,	27	ICC	iTBS (ICC): 0.173	7.8	1) +**23.4%**, 2) **+6.4%**	1.45	Yes
	Sham		+ *r*	iTBS (*r*): **0.097**		Sham: –**2.4%**		
Maeda et al. (2000)	1 Hz,	20	*r*	1 Hz: **0.266**	7	1 Hz: 1) –**16.13%**, 2) –**18.5%**	1.22	No
	10 Hz			10 Hz: **0.26**		10 Hz: 1) –**6.69%**, 2) **−0.22%**		
	20 Hz			20 Hz: **0.543**		20 Hz: 1) **+10.8%**, 2) **+24.58%**		
Vallence et al. (2015)	cTBS	18	ICC	*r* (S1, S2): **0.42**	(S1, S2): 11.6	Inhibition	1.25	No
			+ *r*	*r* (S1, S3): **0.44**	(S1, S3): 22.3			
				SI1mV (ICC): 0.376				
				SI50 (ICC): 0.316				
				150% RMT (ICC): 0.538				
				180% RMT (ICC): 0.539				
Prei et al. (2023)	1 Hz,	30	ICC	1 Hz (*r*): **0.162**	2	No change	2.75	No
	20 Hz		+ *r*	20 Hz (*r*): **0.123**				
				1 Hz (ICC): 0.281				
				20 Hz (ICC): 0.204				
				Median values:				
				1 Hz (r*_*med*_*): 0.322				
				20 Hz (r*_*med*_*): 0.097				
				1 Hz (ICC*_*med*_*): 0.48				
				20 Hz (ICC*_*med*_*): 0.162				
**Studies without reliability value**								
Vernet et al. (2014)	cTBS	10			107	1) **−44%**, 2) **−56%**	1	Yes
Perellón-Alfonso et al. (2018)	iTBS,	20			1	1) **+51%**, 2) **+13.27%**,	1.86	No
	Sham					3) **+53.15%**, 4) **−4.69%**, 5) +**151.75%**		
Bäumer et al. (2003)	1 Hz	11, 5			1, 6	Excitation after 120 min in session 1	0	No
Cohen et al. (2010)	20 Hz	20			0.5	Over day: 1) **+14.94%**, 2) **+14.18%**	0.67	No
	Day, night					Over night: 1) **+13.29%**, 2) **+29.65%**		
Sommer et al. (2002)	2 Hz	6			1	Excitation	n.a.	No
Modugno et al. (2003)	1 Hz	4			0.25	1) **+2.68%**, 2) **−14.29%**	n.a.	No

Overview of the included studies and their methodological properties as well as reliability and aftereffect values. N, number of participants; NN, use of neuronavigation; PAS_25_, paired associative stimulation with 25 ms inter-stimulus interval; Long, long application of the protocol; Short, short application of the protocol; Morning, application of protocol in the morning; Afternoon, application of protocol in the afternoon; iTBS, intermittent theta-burst stimulation; cTBS, continuous theta-burst stimulation; Sham, sham stimulation; day, protocol was applied twice, overday; night, protocol was applied twice, overnight; ICC, intraclass correlation coefficient; *r*, Pearson's *r*; APB, measurement of the abductor pollicis brevis; ADM, measurement of the abductor digiti minimi; Post, reliability of MEPs evoked after rTMS; Post-pre, reliability of MEP aftereffect; AUC, area under the curve; |AUC|, area under the curve of rectified MEPs; (S1, S2), session one in relation to session two; (S1, S3), session one in relation to session three; SI1mV, TMS application with stimulus intensity to evoke 1 mV peak-to-peak MEP amplitude; SI50, TMS application with a stimulus intensity that evokes an MEP halfway between minimal and maximal cortical excitability measured via the input–output curve; 150% RMT, TMS application with supra-threshold stimulus intensity; 180% RMT, TMS application with stimulus intensity evoking maximal MEPs. The column “observed excitability” describes either the mean cortical excitability across all sessions in reference to the baseline activity or if depicted as “1), 2), 3), 4), or 5)” the cortical excitability refers to the change from baseline activity for the respective session. Reliability values printed in bold are depicted in Figure 2, and all bold reliability values except the one for sham stimulation are used in the meta-analysis. Aftereffect values in bold are depicted in Figure 5.

We compiled an overview of reliability values of the different rTMS protocols and interpreted *r* after Cohen (1988), with *r* < 0.1 representing the very small range, 0.1 ≤ *r* < 0.3 small, 0.3 ≤ *r* < 0.5 medium, and 0.5 ≤ *r* ≤ 1 the large range. ICCs were interpreted after Koo and Li (2016), with ICC of < 0.5 being poor, 0.5 ≤ ICC < 0.75 being moderate, 0.75 ≤ ICC < 0.9 being good, and ICC ≥ 0.9 being excellent. To assess whether one rTMS protocol might be superior in reliability to others, we conducted the Kruskal–Wallis test.

### 2.3. Influences of rTMS reliability

In a chi-squared test by Hunter and Schmidt (2000), we assessed the homogeneity of reliability values. To identify whether study-inherent parameters influence rTMS reliability measures, we conducted a random effects regression analysis with Fisher's z-transformed reliability values using the following continuous predictors: “methodological quality”, “year of publication”, “repetition interval of rTMS”, and “sex ratio” as well as categorical predictors “neuronavigation” and “excitability of the protocol”. Hereby, two authors (CK and MO) assessed the methodological quality via the checklist from Chipchase et al. (2012). To ensure the objectivity of the procedure, we calculated Cohen's kappa (κ) for interrater agreement per study with confidence intervals (CI) (Cohen, 1960). A detailed description of how we applied the checklist can be found in the Supplementary material. To assess the influence of the participants' sex, for each study, the sex ratio of the sample was the ratio of the number of female participants per male participants.

To investigate publication bias, we calculated the Rosenthal fail-safe N (Rosenthal, 1979) and conducted a funnel plot for Fisher's z-transformed reliability values and the respective standard errors derived from the sample sizes. By testing the funnel plot for asymmetry (after Egger et al., 1997), we could assess the influence of publication bias on rTMS reliability. We correlated reliability values with the respective year of publication to assess whether more recent research could generate higher reliability of rTMS.

Analyses were run in SPSS (IBM Corp., Version 29) and R (R Core Team, Austria, Version 4.0.5) with the meta package (version 6.2-1) (Schwarzer, 2007). Corresponding syntaxes for the meta-analysis could be found by Field and Gillett (2010).

### 2.4. Aftereffects of rTMS protocols of included studies

We summarized the aftereffects of rTMS, namely, the percent change from baseline MEP to MEP after rTMS stimulation. With the Kruskal–Wallis test, we tested if rTMS protocols had comparable aftereffects. For those studies that did not provide a mean percent change measure, we recalculated mean changes with the provided descriptive data in the manuscripts.

## 3. Results

### 3.1. Study selection and characteristics

**Figure 1:**
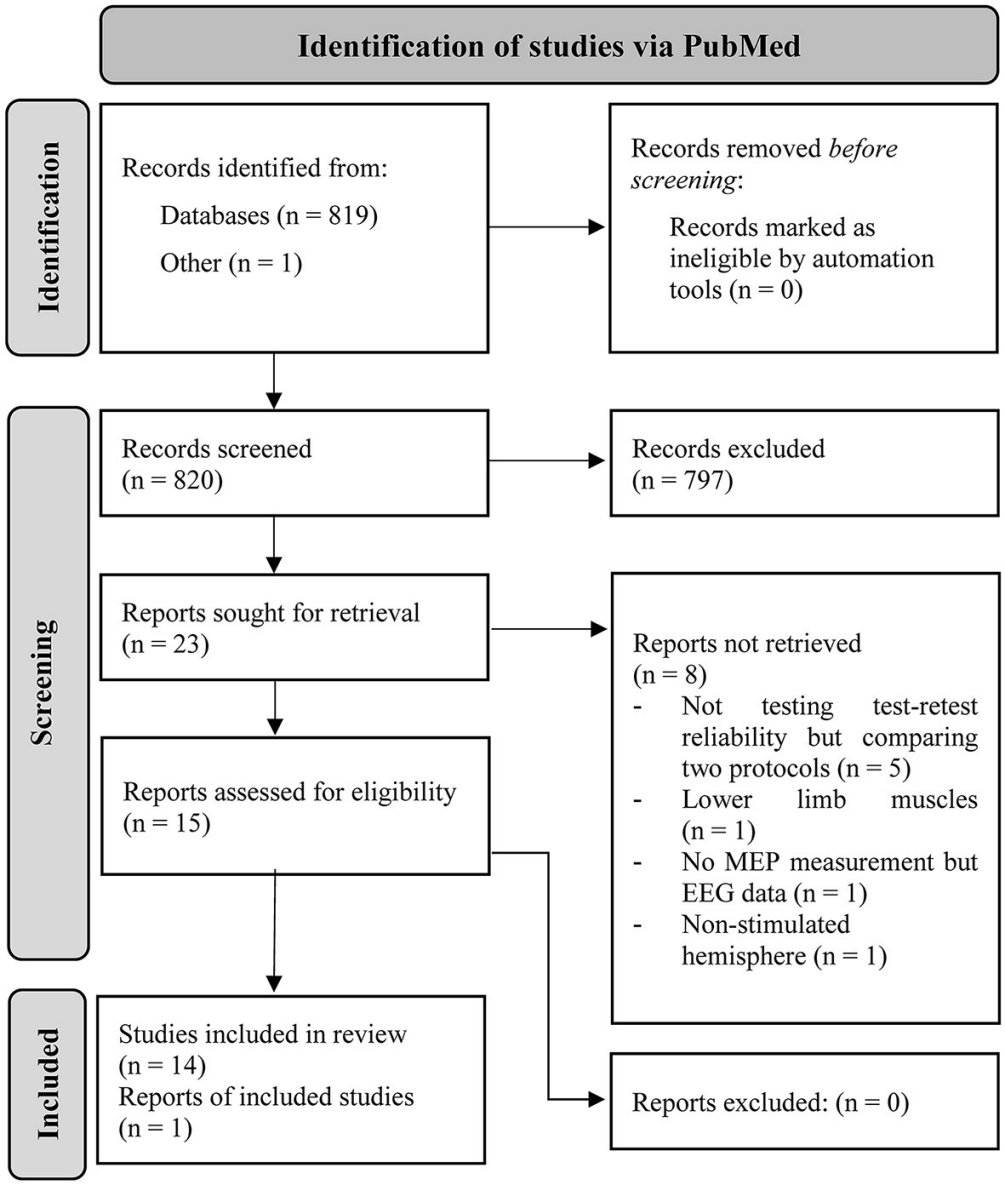
PRISMA workflow of how the articles were identified in PubMed via search string and the procedure of identifying the included studies in this review (Page et al., 2021).

A total of 819 articles resulted from the search in PubMed (latest in March 2023). One preprint was co-authored by CK and included into consideration. In total, 15 articles met the inclusion criteria and were identified as eligible for this review. The detailed procedure can be retraced via the PRISMA flow chart depicted in Figure 1.

The included studies applied continuous rTMS protocols of 1 Hz rTMS (Maeda et al., 2000; Bäumer et al., 2003; Modugno et al., 2003; Prei et al., 2023), 2 Hz rTMS (Sommer et al., 2002), 10 Hz rTMS (Maeda et al., 2000), and 20 Hz rTMS (Maeda et al., 2000; Cohen et al., 2010; Prei et al., 2023). Moreover, test–retest reliability assessments of patterned rTMS protocols were found: iTBS (Hinder et al., 2014; Schilberg et al., 2017; Perellón-Alfonso et al., 2018; Boucher et al., 2021), cTBS (Vernet et al., 2014; Vallence et al., 2015; Jannati et al., 2019; Boucher et al., 2021), and PAS_25_ (Fratello et al., 2006; Sale et al., 2007). Two of these studies included sham stimulation (Perellón-Alfonso et al., 2018; Boucher et al., 2021).

The mean sample size of studies was *n* = 17 (range 4–30, total: 291), with overall 138 women, 143 men, and 10 not applicable. Participants had a mean age of 27 years (range: 18–65 years). The mean test–retest intervals were 13.41 days (range: 6 h to 107 days).

Fratello et al. (2006) reported ICCs for the muscle at which the representative cortical spot was stimulated, i.e., abductor pollicis brevis (APB), as well as one other muscle, the abductor digiti minimi (ADM). For both muscles, the authors computed ICCs with the respective post-rTMS measure as well as with the rTMS aftereffects (post-pre). Sale et al. (2007) computed ICCs for a long and a short application of PAS_25_ as well as for the groups that attended sessions in the morning and afternoon, respectively. Boucher et al. (2021) reported *r* values for the overall measurement of iTBS, cTBS, sham, and ICCs for 5, 10, 20, 30, 50, and 60 min after rTMS application. Hereby, we summarized the ICCs by reporting the minimal and maximal ICC of the respective procedures. Jannati et al. (2019) assessed the reliability values from the area under the curve (AUC) of elicited MEPs as well as for the area under the curve of rectified MEPs (|AUC|). Vallence et al. (2015) reported *r* values for two sessions each, i.e., sessions 1 and 2 as well as sessions 1 and 3. The authors also conducted ICC for rTMS aftereffect assessment at stimulus intensities that elicited peak-to-peak MEP amplitudes of 1 mV ± 0.15 mV (SI1 mV). Additionally, ICC values for stimulus intensities measured via the input–output curve (IO curve) that evoked MEP sizes halfway between minimal and maximal cortical excitability (SI50), supra-threshold stimulus intensities (150% RMT), and stimulus intensities evoking maximal MEPs (180% RMT) were conducted. Prei et al. (2023) reported *r* as well as ICCs of mean and median MEPs over the whole measurement and the respective quarters, whereby we report the overall measures. Table 1 gives an overview of the methodological and result parameters of interest.

### 3.2. Test–retest reliability

Five of the 15 studies did not report any reliability value but only measures of variance (Sommer et al., 2002; Bäumer et al., 2003; Modugno et al., 2003; Cohen et al., 2010; Vernet et al., 2014). One study only conducted ICCs for baseline MEPs but not for MEPs after the rTMS procedure (Perellón-Alfonso et al., 2018). Therefore, these six studies were excluded from the subsequent analyses of reliability, resulting in nine evaluable studies. Both ICC and *r* values were reported in five studies; one publication conducted only *r* and three only ICCs. We preferably extracted *r* values instead of ICC as well as the overall reliability for a respective protocol within the studies. Whenever reliability values from the same sessions were calculated, we chose mean MEP amplitudes instead of median (Prei et al., 2023) and values derived from the muscle whose cortical representation was stimulated (Fratello et al., 2006) as well as the non-rectified derived parameter (Jannati et al., 2019). Five out of nine studies reported the reliability of aftereffect measures, one reported the reliability of post-rTMS measures (Hinder et al., 2014), two did not state which measure they chose (Sale et al., 2008; Boucher et al., 2021), and one reported both the post and the post-pre measures (Fratello et al., 2006). The highest value was used (post-measure) obtained from the study of Fratello et al. (2006) for depiction and meta-analysis in order to gain the most information out of the publication bias investigation.

**Figure 2:**
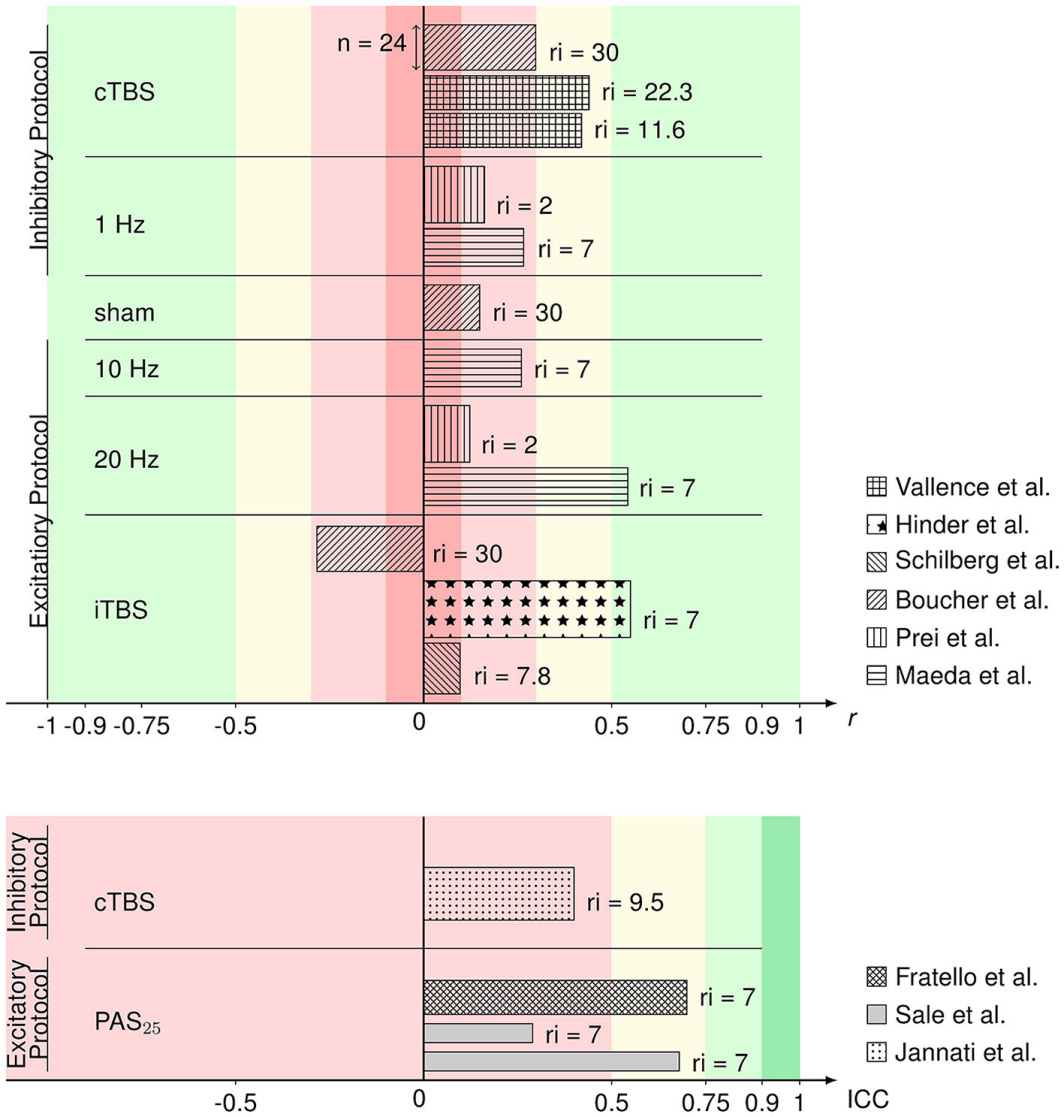
Bar graph of Pearson's *r* **(upper)** and ICC values **(lower)** of the rTMS protocols from the respective articles identified as eligible and comparable in this review. Reliability values are sorted by protocol and the respective excitability. Patterns of the bars represent in which article the respective values were published. The width of the bars represents the sample size within the respective studies. On the right side of the bars, the (mean or minimum of) number of days between repetition of the rTMS protocol (repetition interval; ri) are depicted. Colors represent the interpretation of *r* (Cohen, 1988) and ICC (Koo and Li, 2016) values as negligible (darker red), poor/low (lighter red), medium/moderate (yellow), good/large (lighter green), or excellent (darker green).

Figure 2 gives an overview of the included comparable values from evaluable studies combined with a representation of the number of participants (sample size) per experiment and the time interval of rTMS repetition. ICCs in this overview of reliability measures ranged from 0.29 to 0.7 and *r* from 0.097 to 0.55 with one iTBS reliability value being in the negative range (−0.284). Reliabilities did not differ between rTMS protocols in analysis via the Kruskal–Wallis test ($\chi_{\left(5\right)}^{2}$ = 5.292, *p* = 0.381). The exact values can be found in Table 1. According to the classification after Cohen (1988), rTMS reliability yields small to medium effect sizes. The reliabilities of the 20 Hz rTMS protocol (Maeda et al., 2000) and one iTBS protocol (Hinder et al., 2014) show large effect sizes (*r* = 0.543 and *r* = 0.55, respectively). ICCs also range from poor to moderate reliability values (Koo and Li, 2016). 9 out of 15 rTMS reliability values were interpreted as small or poor, 4 out of 15 as medium or moderate and 2 out of 15 as large effects sizes.

### 3.3. Influences of rTMS reliability

**Figure 3:**
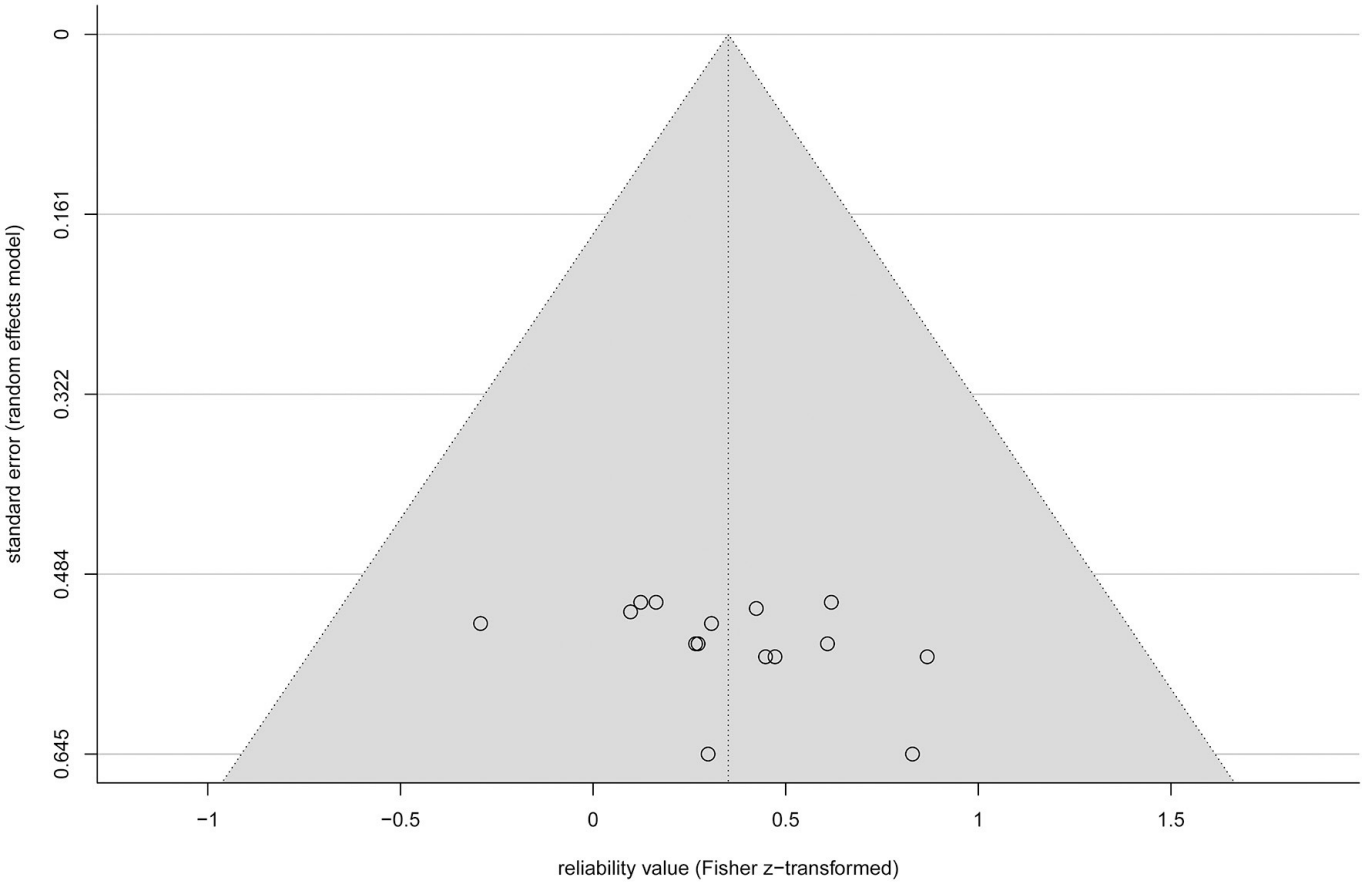
Funnel plot of the standardized effect sizes (Fisher z-transformed values of *r* and ICC) of the eligible studies plotted against their standard error from the random effects model (circles). The diagonal lines (gray background) depict the 95% confidence interval.

**Figure 4:**
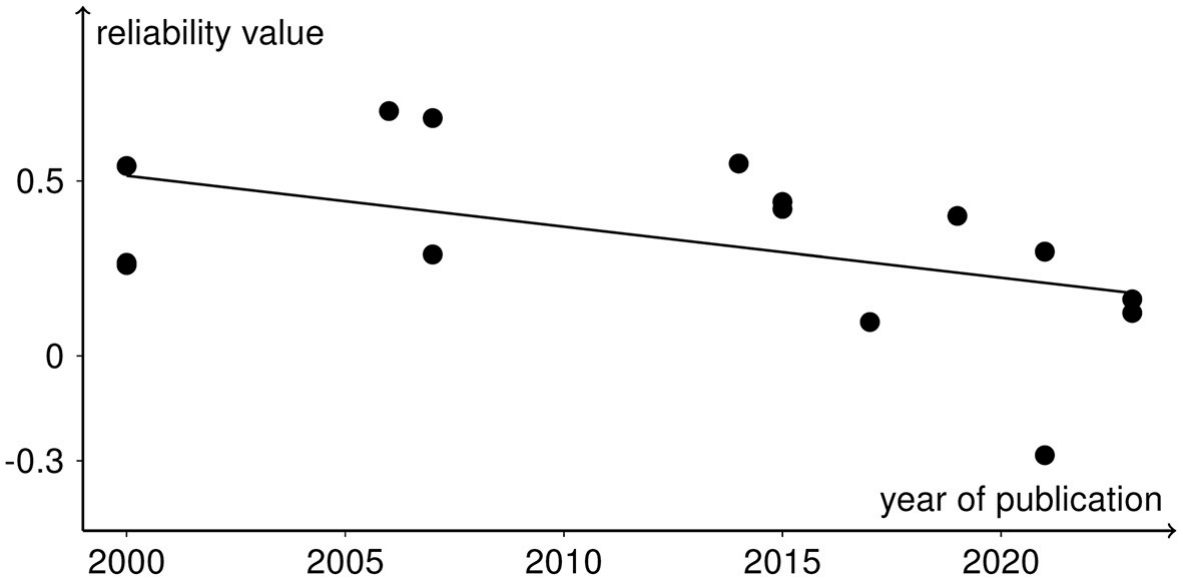
Reliability values as black dots included in the meta-analysis with the year of publication of the respective studies in a scatter plot. The negative trend from the correlation analysis is depicted as the black line.

All analyses include both *r* and ICC values because otherwise not all rTMS protocols would be covered. No significant heterogeneity between study reliabilities was found via the chi-squared test ($\chi_{\left(14\right)}^{2}$ = 20.945, *p* = 0.103). The random effects regression analysis revealed an overall mean Fisher's z-transformed reliability value of 0.315 (95% CI range: 0.168 to 0.449) that is significantly different from zero (*t*_(15)_ = 4.45, *p* = 0.001) in a model without any predictors. By including the continuous and categorical predictors, none of them showed a significant influence (continuous: *t*_(7)_ < 1, *p* > 0.442 and categorical: $\chi_{\left(1\right)}^{2}$ < 1, *p* > 0.714). Methodological quality assessed with the checklist by Chipchase et al. (2012) ranged from 30.8 to 55.8% and interrater agreement from 0.65 (CI range: 0.5 to 0.82) to 1 (CI range: 1 to 1). Detailed ratings can be found in Supplementary Tables S1, S2. Additionally, the goodness of fit of the random effects regression analysis was not significant ($\chi_{\left(8\right)}^{2}$ = 7.935, *p* = 0.44). The Rosenthal fail-safe N was 164. The funnel plot, representing Fisher's z-transformed reliability values on the x-axis and the standard errors from the random effects model on the y-axis, is depicted in Figure 3. With a test for funnel plot asymmetry (after Egger et al., 1997), no significant skewedness was identified (*t*_(13)_ = 1.42, *p* = 0.179). Correlating reliability values with their respective year of publication revealed a negative but non-significant result (*r* = −0.475, *p* = 0.073), which is shown in Figure 4.

### 3.4. Aftereffects of rTMS procedures of included studies

**Figure 5:**
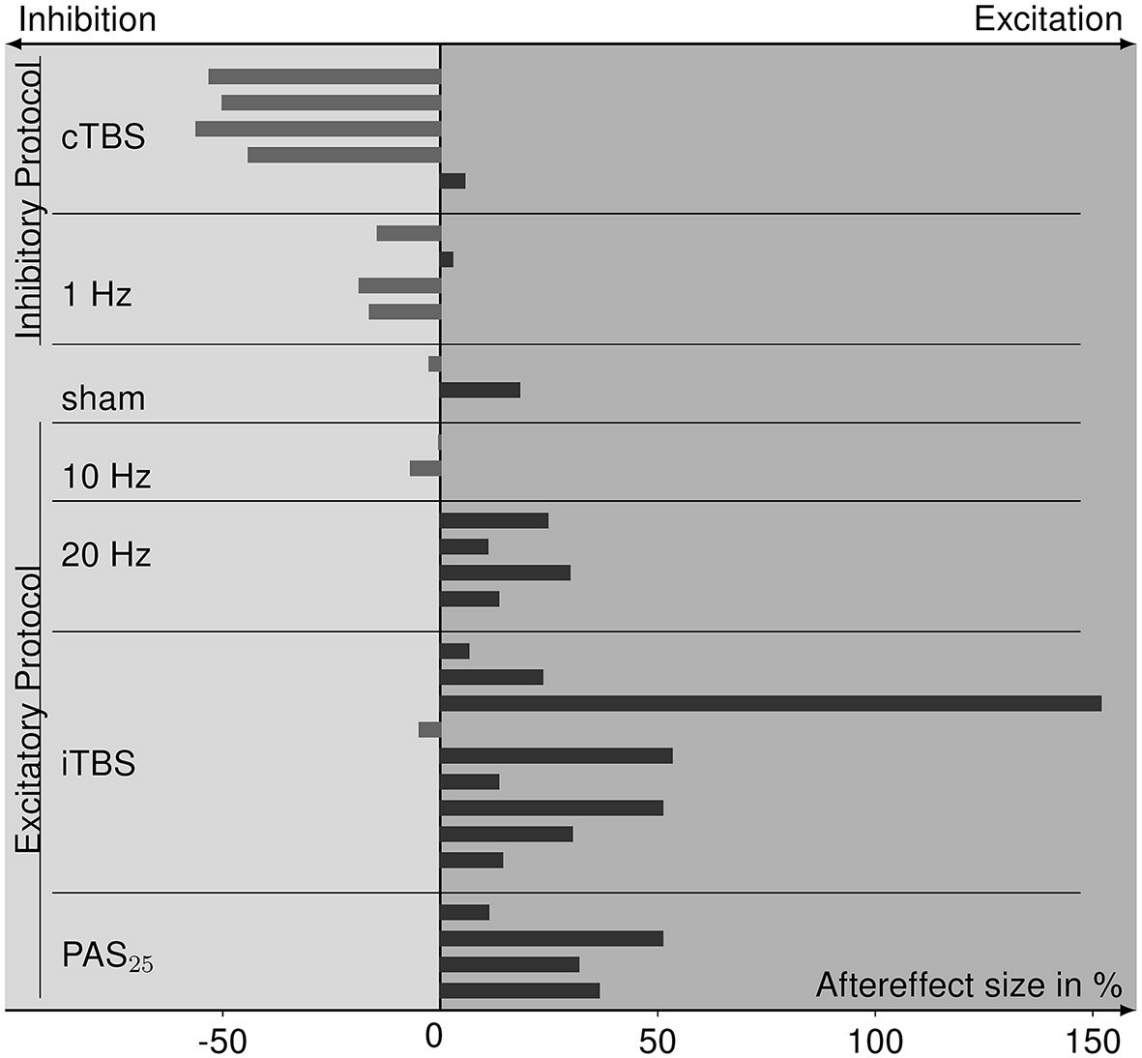
Bar graph of aftereffect sizes, i.e., the percent change of MEPs after rTMS relative to pre-rTMS (baseline) MEPs of the data collected from the eligible studies and sorted for each rTMS protocol. Protocols are grouped for inhibitory and excitatory effect expectations according to the TMS-protocol-dependent inhibition-excitation heuristic. Positive aftereffect sizes indicate cortical excitation after rTMS stimulation compared to baseline, whereas negative aftereffect sizes refer to cortical inhibition.

From four experiments (Sommer et al., 2002; Bäumer et al., 2003; Vallence et al., 2015; Prei et al., 2023), we were not able to retrieve information on aftereffect sizes, resulting in 11 studies whose data can be included in a summary of aftereffects (for values see Table 1). The size and direction of aftereffects for each rTMS protocol are depicted in Figure 5. The protocols known to primarily evoke cortical inhibition (cTBS and 1 Hz) mainly show the inhibitory effects of MEP amplitudes after rTMS application. Sham stimulation has both aftereffect directions. Excitatory protocols (20 Hz, iTBS, PAS_25_) mostly evoke cortical excitation, except for the 10 Hz protocol that has inhibitory tendencies. Analysis via the Kruskal–Wallis test revealed differences in aftereffects between the rTMS protocols ($\chi_{\left(6\right)}^{2}$ = 20.762, *p* = 0.002), whereby cTBS had significantly lower effects than 20 Hz rTMS (*p* = 0.005), iTBS (*p* < 0.001), and PAS_25_ (*p* = 0.001), and 1 Hz rTMS had significantly lower aftereffects than 20 Hz rTMS (*p* = 0.033), iTBS (*p* = 0.008), and PAS_25_ (*p* = 0.008). Nevertheless, within excitatory or inhibitory protocols, there were no differences in aftereffects (*p* > 0.054).

## 4. Discussion

This study provides an overview of test–retest reliability of rTMS procedures. We assessed whether we could identify a procedure to be most effective and reliably induce neuroplastic changes in the brain. We identified whether parameters inherent to the respective publication influenced reliability values. To estimate the representativity of reliability values, we checked for publication bias.

A total of 15 studies were found that assessed whether rTMS protocols evoke repeatable cortical reactions over time, i.e., their test–retest reliability. Pearson's *r* and ICC values were interpreted as small in nine and medium in four studies. Only the reliabilities of the 20 Hz rTMS protocol (Maeda et al., 2000) and of one iTBS protocol (Hinder et al., 2014) are to be interpreted as large according to Cohen (1988). Nevertheless, there is no evidence to favor one particular rTMS protocol based on higher reliability values since no value showed a significant difference from the mean reliability in the Kruskal–Wallis test. Moreover, the chi-squared test did not confirm a difference between reliabilities, which additionally approves the joint analysis of Pearson's *r* and ICC values. One negative reliability value (*r* = −0.284) for iTBS was identified, meaning that the participants' first application of iTBS resulted in a facilitatory effect, whereas the second application had an inhibitory effect and vice versa (Boucher et al., 2021). The two other experiments assessing iTBS reliability did not show such a relation.

Parameters that depend on the publication, i.e., methodological quality of the study and year of publication, and on experimental design, i.e., excitability of protocol, use of neuronavigation, sex ratio of the sample, and rTMS repetition interval, had no influence on the reliability of rTMS. Publication bias did not affect the present reliability values indicated by the funnel plot and asymmetry test, which thus strengthens the credibility of these reliability estimates. Nevertheless, the funnel plot depicts that all reliability values are associated with relatively high standard errors, which are derived from the sample sizes of the studies. The Rosenthal fail-safe N of 164 indicates that, if 164 unpublished studies had a non-significant reliability parameter, the estimated overall reliability would turn significantly different from zero to non-significant. By correlating reliability values with the respective year of publication, we identified a negative trend, indicating that, in the current research study, reliability values decrease. This could be either due to higher publication bias in past publications or due to increasing variability perhaps induced by the plurality of setup equipment and higher accuracy of measures, e.g., TMS stimulators, coils, and electrodes. Moreover, higher objectivity of assessment might lead to reduced reliability values. Thus, further studies are needed with higher sample sizes and a systematic investigation of reliability to strengthen the assumption that no publication bias is present in rTMS reliability studies.

rTMS protocols of our included studies followed the lofi-hife heuristic and association with respective inhibition or facilitation effects. Most of the protocols mainly evoking inhibitory neuronal effects resulted in reduced MEPs after stimulation, and the protocols primarily having excitatory effects produced higher MEPs after stimulation. Only the 10 Hz rTMS protocol (Maeda et al., 2000) shows contrary results. The Kruskal–Wallis test confirmed that inhibitory rTMS protocols had significantly lower aftereffects than excitatory protocols, whereby 10 Hz rTMS did not differ significantly from both inhibitory and excitatory protocols.

Identification of the rTMS protocol with the most reliable and effective outcome cannot be provided currently. Since reliability values did not differ between protocols and also within excitatory and inhibitory protocols, aftereffects were comparable, no superiority of certain protocols can be proven. Descriptively, cTBS seems to have better inhibitory aftereffects and reliability than 1 Hz rTMS. For excitatory protocols, iTBS can induce descriptively higher aftereffects than PAS_25_, 20 Hz and 10 Hz rTMS, but also varies more in reliability.

In contrast to baseline spTMS test–retest reliability values reaching ICCs of 0.86 (Pellegrini et al., 2018), rTMS reliability values tend to be smaller. It is important to note that, to assess rTMS reliability, both rTMS and spTMS need to be applied. Thus, variability in both measures adds up to the resulting rTMS aftereffect reliability value. To assess cortical excitability, IO curves cover the whole spectrum best. Nonetheless, to gain clear insights into brain functions and effective treatment of disorders by rTMS, reliable measurements are necessary at best with reliability values in the large range. On the one hand, this is achieved by identifying and eliminating or controlling parameters that influence the variability of spTMS and rTMS. On the other hand, personalization of applications can be an effective method (Schoisswohl et al., 2021).

Many parameters that influence the variability of spTMS and rTMS are already identified, e.g., stimulation intensity and number of applied pulses (Pascual-Leone et al., 1994; Fitzgerald et al., 2002; Peinemann et al., 2004; Lang et al., 2006), pulse form (Arai et al., 2005), time of day (Sale et al., 2008), subject-related factors such as age (Rossini et al., 1992; Pitcher et al., 2003; Todd et al., 2010; Cueva et al., 2016), genetic factors (Cheeran et al., 2008; Di Lazzaro et al., 2015), and changes in motor activation state (Huang et al., 2008; Iezzi et al., 2008; Goldsworthy et al., 2014). The identified studies assessing rTMS reliability show that, with higher stimulus intensities, cortical inhibition increases during cTBS and perceived stress correlated with larger aftereffects (Vallence et al., 2015). A 20 Hz rTMS application with a night in between resulted in higher aftereffects than stimulation overday (Cohen et al., 2010). Influences on the reliability of rTMS procedures were assessed by Jannati et al. (2019), who showed in an exploratory analysis that age and genotype had an influence on the reliability of cTBS aftereffects. Sale et al. (2007) revealed that PAS_25_ assessment in the afternoon is more reliable than in the morning. To systematically investigate which parameters affect the reliability of rTMS, further studies are needed with more power and randomized and controlled experimental design. A meta-analysis is hereby not sufficient to extract dependable information. To generate comparable data on rTMS reliability, future studies should report both Pearson's *r* value and ICC with corresponding confidence intervals as well as the model that the ICC calculation was based on. It should also be established to compute and report both reliability of post-rTMS measures and rTMS aftereffects. Further research on the variability and test–retest reliability of rTMS procedures is needed to identify factors that improve rTMS reliability and estimate the maximal reliability values achievable.

Although the induction of expected inhibitory or facilitatory aftereffects by rTMS protocols seems to succeed, high inter- and intra-individual variabilities dominate the results of rTMS experiments (Schilberg et al., 2017), even when controlling for most influencing parameters. In an experiment by Hamada et al. (2013), 50% of the aftereffect variation after TBS was predicted by a marker for late I-wave recruitment, which is discussed to be a mechanism of neuromodulation (Di Lazzaro et al., 2004). Thus, the other 50% of the variation is not yet explainable, also being not related to age, gender, time of day, and baseline MEP sizes (Hamada et al., 2013). It raises the question whether rTMS in general leads to the induction of variability in the neuronal responses and thus does not achieve exclusively LTP- or LTD-like plasticity effects. Thus, investigations of variability, e.g., the coefficient of variation in addition to the mean evoked responses could provide explanations. Additionally, all-encompassing sham conditions could reveal unbiased aftereffects.

Another approach to identifying reasons for the variability of rTMS is to investigate patients. For example, patients with Alzheimer's disease characterized by neuronal degeneration and rigidity show higher reliability of rTMS compared to healthy controls (Fried et al., 2017). One explanation might be that, in Alzheimer's patients, impairments of cortical plasticity can lead to omitted rTMS aftereffects (Di Lorenzo et al., 2020). Nevertheless, there is also evidence that patients with more severe Alzheimer's disease markers show higher inhibitory aftereffects after 1 Hz rTMS (Koch et al., 2011), which might indicate that neuronal rigidity can be altered by the induction of variability with rTMS. Thus, interpretive approaches should be taken with caution.

The present systematic review and meta-analysis address the test–retest reliability of rTMS on healthy individuals, and derived findings cannot be transferred to other populations, such as patient groups, or other applications, e.g., stimulation over other cortices than the motor cortex. Participants from the included studies were often right-handed and accordingly did not show a representative sample. Because only a few studies contributed to the analysis of reliability values, the results cannot be generalized and need to be interpreted with caution. Additionally, although eliciting MEPs is a common procedure to investigate cortical excitability, it still represents an indirect measure. There are other markers that hold the potential to estimate reactions of the brain to rTMS in a more direct way, yet, to date, they are studied less frequently and prone to artifacts and noise.

Test–retest reliability of rTMS in the identified studies is mainly small to moderate, with overall scarce experimental assessment. Aftereffects of rTMS protocols mainly followed the respective inhibition or excitation expectation. No protocol is to be favored based on our findings of reliability values and aftereffect sizes. However, the generalizability remains questionable because of limited comparable data. By reporting ICC as well as Pearson's *r* values of both post-rTMS and aftereffect measures, studies examining test–retest reliability can contribute to comparability. Additionally, the application of spTMS should be equal, e.g., by assessing the IO curves of MEPs. In general, the variability of NIBS outcomes is mirrored in its reliability. Influential factors of both spTMS and rTMS need to be systematically investigated to achieve high and reliable rTMS aftereffects. To establish rTMS procedures in the clinical everyday use of disorder treatment, higher reliability is necessary. With this overview, scientists and clinicians can estimate and compare the size and reliability of the aftereffects of rTMS based on current data.

## Data availability statement

The original contributions presented in the study are included in the article/Supplementary material, further inquiries can be directed to the corresponding author.

## Author contributions

CK, SS, and MS contributed to the review concept and design. CK performed the literature search and conducted data analysis and visualized the study and drafted the manuscript. CK and MO reviewed included studies and examined the studies' methodological quality. CK, MO, FS, KL, WS, WM, SS, and MS revised the manuscript. All authors have read and approved the final version. All authors contributed to the article and approved the submitted version.
