# Supplementary material for: Retest reliability of repetitive transcranial magnetic stimulation over the healthy human motor cortex: a systematic review and meta-analysis
Source: Front Hum Neurosci. 2023 Sep 13;17:1237713. doi: 10.3389/fnhum.2023.1237713 (PMC10525715; doi:10.3389/fnhum.2023.1237713)
Supplement: Supplementary file 1 [file Data_Sheet_1.docx]

Supplementary Material

Retest reliability of repetitive transcranial magnetic stimulation over the healthy human motor cortex: a systematic review and meta-analysis

Carolina Kanig^1,2*^, Mirja Osnabruegge^1,2^, Florian Schwitzgebel^3^, Karsten Litschel^3^, Wolfgang Seiberl^4^, Wolfgang Mack^1^, Stefan Schoisswohl^1,2^, Martin Schecklmann^2^

***Correspondence:** Carolina Kanig
 Email: [carolina.kanig@unibw.de](mailto:carolina.kanig@unibw.de)

# Supplementary Data

## Methodological quality of included studies

We assessed the methodological quality of the studies with the checklist by Chipchase et al. (2012). From the originally 30 items, with three only assessible for paired-pulse TMS, author CK and MO classified the item “subjects prescribed medication” from the checklist as too little different from the item “use of CNS active drugs” and merged these into one item resulting in 26 assessed items for single pulse TMS measurements. For rTMS procedures there are currently no factors included in the checklist. Each item was independently checked whether it was experimentally “controlled” , descriptively “reported” or not reported at all – “missing”, whereby “controlled” items are also “reported”. “Controlled” means that a factor was excluded, e.g., by exclusion criteria, or a factor was balanced e.g., “gender of subjects”, or used as a covariate in data analysis. We assume that the term “gender” in the checklist is mistakenly used for “sex”, because the female cycle can have an influence on cortical excitability (Inghilleri et al., 2004). Although the checklists authors state otherwise, we considered the item “gender of subjects” as controllable, i.e., by inserting the factor in as a covariate in the analyses and the item “level of relaxation of muscles other than those being tested” as reportable, i.e., with visual monitoring. The percentages for the “reported” items (Percent_R_) were calculated by dividing the total number of “reported” items by the total number of the 26 items per study and rater (CK and MO). Equally, percentages of “controlled” items of the checklist (Percent_C_) were calculated equivalent to Percent_R_. A total percentage (Percent_T_) was calculated by calculating the mean of Percent_C_ and Pecent_R_ of rater 1. The total percentage was considered to represent the overall methodological quality and also used as a covariate in the meta-analysis for accounting of methodological differences between studies. For interrater agreement we calculated Cohen’s kappa (κ) per study with confidence intervals (CI) (Cohen, 1960).

## Results

Percent_R_ was on average 73.3% in a range from 61.5% to 84.6%. Percent_C_ was in the range from 0% to 38.5% resulting in an average of 18.5%. The mean Percent_T_ was 45.8%. Detailed percentages of both raters as well as Cohens kappas for interrater agreement are specified in **Table S1**. Absolute frequencies for each item that was rated as “reported” and “controlled” for all studies are depicted in **Table S2**. Ten out of 15 studies “controlled” for “time between days of testing” and “size of unconditioned MEP” and seven for “time between MEP trials”, being the most “controlled” items. Items that often were neither “reported” nor “controlled”, i.e. “missing” were “level of relaxation of muscles other than those being tested” in 14 out of 15 studies, “history of specific repetitive motor activity” in 13 studies and “any medical condition” in 11 studies. ᴋ ranged from 0.65 to 1.

# Supplementary Figures and Tables

## Supplementary Tables

**Supplementary Table S1.** Overview of Cohens kappas and Chipchase percentages of included studies. This table gives an overview of included studies depicting Cohens kappas (κ), 95% confidence intervals (CI) of ᴋ and the percentages of experimentally controlled items assessed by rater 1 (Percent**_C1_**) and descriptively reported items assessed by rater 1 (Percent**_R1_**) as well as percentages of controlled items assessed by rater 2 (Percent**_C2_**) and descriptively reported items assessed by rater 2 (Percent**_R2_**). Percent_T_ are the total percentages per study of rater 1.

| **Study Authors and Year** | **Percent_R1_** | **Percent_C1_** | **Percent_R2_** | **Percent_C2_** | **Percent_T_** | **κ** | **CI** |
| --- | --- | --- | --- | --- | --- | --- | --- |
| Fratello et al. (2006) | 76.9% | 26.9% | 76.9% | 26.9% | 51.9% | 1 | 1 – 1 |
| Sale et al. (2007) | 80.8% | 30.8% | 80.8% | 30.8% | 55.8% | 1 | 1 – 1 |
| Boucher et al. (2021) | 76.9% | 7.7% | 73.1% | 11.5% | 42.3% | 0.85 | 0.67 – 1 |
| Vernet et al. (2014) | 84.6% | 11.5% | 84.6% | 11.5% | 48.1% | 1 | 1 – 1 |
| Jannati et al. (2019) | 80.8% | 15.4% | 80.8% | 15.4% | 48.1% | 1 | 1 – 1 |
| Hinder et al. (2014) | 73.1% | 15.4% | 73.1% | 15.4% | 44.2% | 1 | 1 – 1 |
| Perellón-Alfonso et al. (2018) | 69.2% | 38.5% | 69.2% | 38.5% | 53.9% | 1 | 1 – 1 |
| Schilberg et al. (2017) | 80.8% | 30.8% | 80.8% | 30.8% | 55.8% | 1 | 1 – 1 |
| Vallence et al. (2015) | 65.4% | 23.1% | 65.4% | 23.1% | 44.2% | 1 | 1 – 1 |
| Bäumer et al. (2003) | 76.9% | 19.2% | 76.9% | 19.2% | 48.1% | 1 | 1 – 1 |
| Cohen et al. (2010) | 69.2% | 11.5% | 65.4% | 11.5% | 40.4% | 1 | 1 – 1 |
| Maeda et al. (2000) | 73.1% | 3.8% | 73.1% | 7.7% | 38.5% | 0.92 | 0.74 – 1 |
| Sommer et al. (2002) | 57.7% | 11.5% | 57.7% | 15.4% | 34.6% | 0.94 | 0.71 – 1 |
| Modugno et al. (2003) | 61.5% | 0.0% | 61.5% | 0.0% | 30.8% | 1 | 1 – 1 |
| Prei et al. (2023) | 76.9% | 23.1% | 76.9% | 26.9% | 50.0% | 0.65 | 0.5 – 0.82 |

**Supplementary Table S2.** Overview of items from the Chipchase (2012) checklist with the respective absolute frequencies of studies “reporting” and “controlling” for each item. The assessments of both raters are depicted individually.

|  | **Rater 1** | | **Rater 2** | |
| --- | --- | --- | --- | --- |
|  | **controlled** | **reported** | **controlled** | **reported** |
| **Participant factors** | | | | |
| Age of subjects | 1 | 15 | 1 | 15 |
| Gender of subjects | 4 | 14 | 4 | 14 |
| Handedness of subjects | 6 | 12 | 6 | 12 |
| Use of CNS active drugs | 1 | 3 | 1 | 3 |
| Presence of neurological/psychiatric disorders when studying healthy subjects | 2 | 11 | 2 | 11 |
| Any medical condition |  | 4 |  | 4 |
| History of specific repetitive motor activity | 1 | 2 | 1 | 2 |
| **Methodological factors** | | | | |
| Position and contact of EMG electrodes | 2 | 13 | 2 | 13 |
| Amount of relaxation/contraction of target muscles | 7 | 14 | 6 | 14 |
| Prior motor activity of the muscle to be tested | 3 | 5 | 3 | 5 |
| Level of relaxation of muscles other than those being tested | 1 | 1 | 1 | 1 |
| Coil type (size and geometry) | 0 | 14 | 0 | 14 |
| Coil orientation | 0 | 13 | 0 | 13 |
| Direction of induced current in the brain | 0 | 8 | 0 | 8 |
| Coil location and stability (with or without neuronavigation system) | 2 | 12 | 3 | 12 |
| Type of stimulator used |  | 15 |  | 15 |
| Stimulation intensity | 5 | 14 | 5 | 14 |
| Pulse shape (mono/bi) |  | 10 |  | 10 |
| Determination of optimal hotspot | 1 | 13 | 1 | 13 |
| Time between MEP trials | 8 | 14 | 7 | 14 |
| Time between days of testing | 13 | 15 | 10 | 15 |
| Subject attention (level of arousal) during testing | 1 | 5 | 1 | 5 |
| Method for determining threshold (active/resting) | 1 | 15 | 1 | 15 |
| Number of MEP measures made | 3 | 15 | 3 | 15 |
| **Analytical factors** | | | | |
| Method for determining MEP size during analysis | 2 | 14 | 2 | 15 |
| Size of unconditioned MEP | 10 | 14 | 10 | 14 |

References

Chipchase, L., Schabrun, S., Cohen, L., Hodges, P., Ridding, M., Rothwell, J., et al. (2012). A checklist for assessing the methodological quality of studies using transcranial magnetic stimulation to study the motor system: an international consensus study. *Clin Neurophysiol* 123, 1698–1704. doi: 10.1016/j.clinph.2012.05.003

Cohen, J. (1960). A Coefficient of Agreement for Nominal Scales. *Educational and Psychological Measurement* 20, 37–46. doi: 10.1177/001316446002000104

Inghilleri, M., Conte, A., Currà, A., Frasca, V., Lorenzano, C., and Berardelli, A. (2004). Ovarian hormones and cortical excitability. An rTMS study in humans. *Clin Neurophysiol* 115, 1063–1068. doi: 10.1016/j.clinph.2003.12.003
